# Supplementary material for: Decoupling between the genetic potential and the metabolic regulation and expression in microbial organic matter cleavage across microbiomes
Source: Microbiol Spectr. 2024 Mar 21;12(5):e03036-23. doi: 10.1128/spectrum.03036-23 (PMC11210215; doi:10.1128/spectrum.03036-23)
Supplement: Supplemental figures and tables — Figure S1 to S14; Table S1 to S3. [file spectrum.03036-23-s0008.pdf]

Supplementary information for

Decoupling between genetic potential and the metabolic regulation and expression in microbial organic matter cleavage across microbiomes

Zihao Zhao[1*], Federico Baltar[1], Gerhard J. Herndl[1, 2, 3*]

[1] Department of Functional and Evolutionary Ecology, University of Vienna, Djerassiplatz 1, A-1030 Vienna, Austria

[2] NIOZ, Department of Marine Microbiology and Biogeochemistry, Royal Netherlands Institute for Sea Research, Den Burg, The Netherlands

[3] Vienna Metabolomics Center, University of Vienna, Djerassiplatz 1, A-1030 Vienna, Austria

*Correspondence to: zihao.zhao@univie.ac.at; gerhard.herndl@univie.ac.at

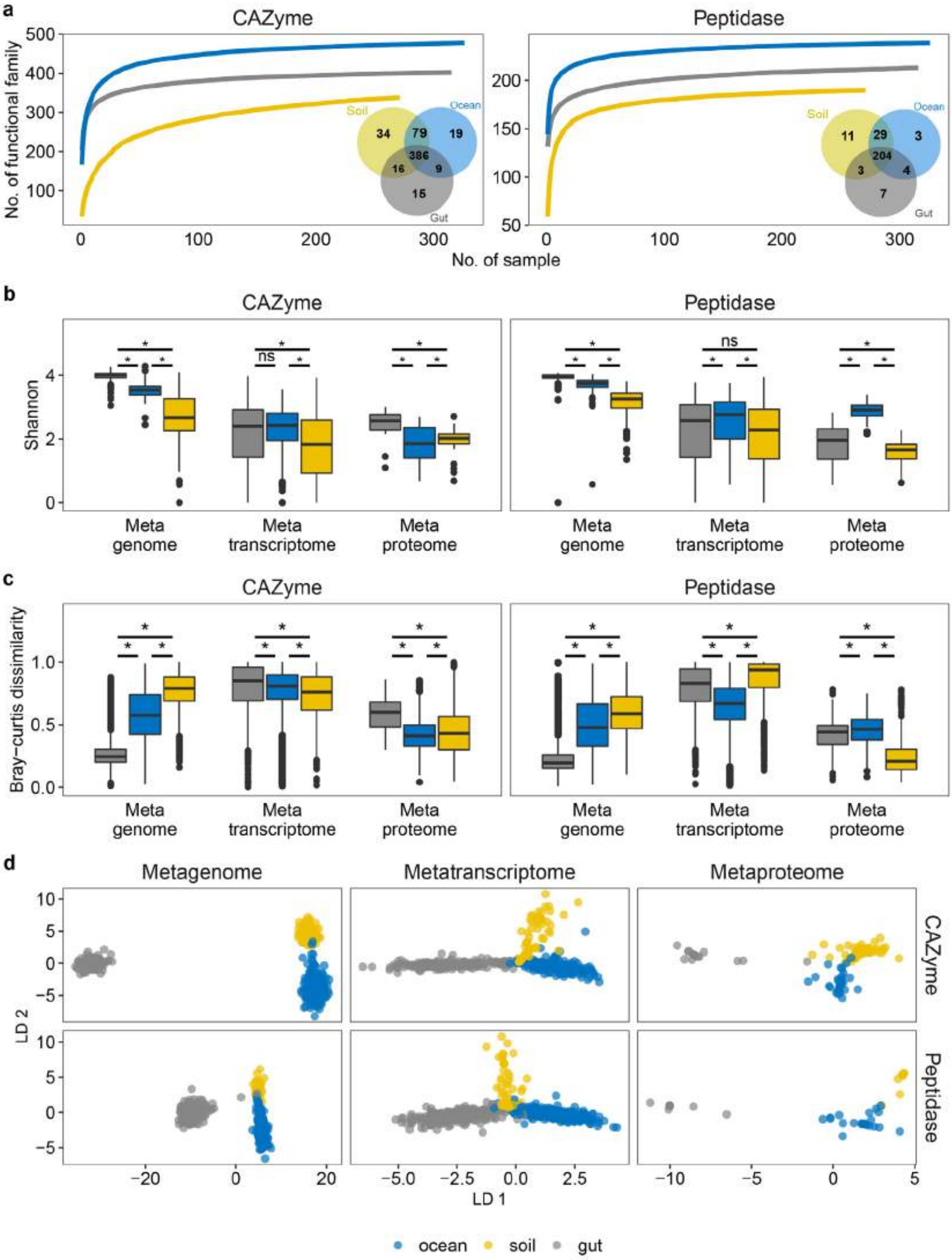

a
CAZyme
Peptidase
No. of functional family
No. of sample
Soil
Ocean
Gut
34
79
19
386
16
9
15
11
29
3
204
3
4
7
b
Shannon
c
Bray-curtis dissimilarity
Meta genome
Meta transcriptome
Meta proteome
ns
d
Metagenome
Metatranscriptome
Metaproteome
CAZyme
Peptidase
LD 1
LD 2
ocean
soil
gut

Fig. S1 | Multi-omics profiles of cytoplasmic CAZymes and peptidases indicating distinct distribution patterns across microbiomes. a, Venn diagram of CAZyme and peptidase families identified by metagenomic assembly, numbers show the unique/shared subfamilies in each microbiome. **b** and **c**, Shannon index-based α-diversity and Bray-Curtis dissimilarity-based β-diversity for secretory CAZymes and peptidases in multi-omics datasets. **d,** Linear discriminate analysis of secretory CAZymes and peptidases in multi-omics datasets. Statistics are based on Wilcoxon test (*, $p<0.05$; ns, not significant)

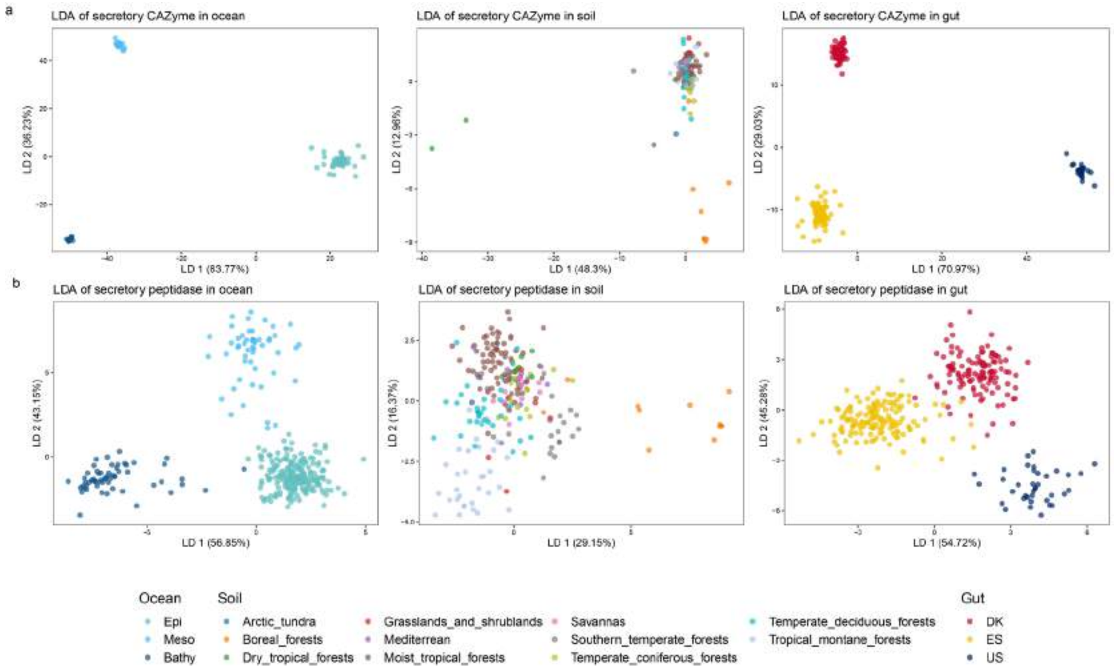

Fig. S2 | Linear discriminate analysis of genes encoding secretory CAZymes and peptidases in the ocean, soil and human gut microbiome.

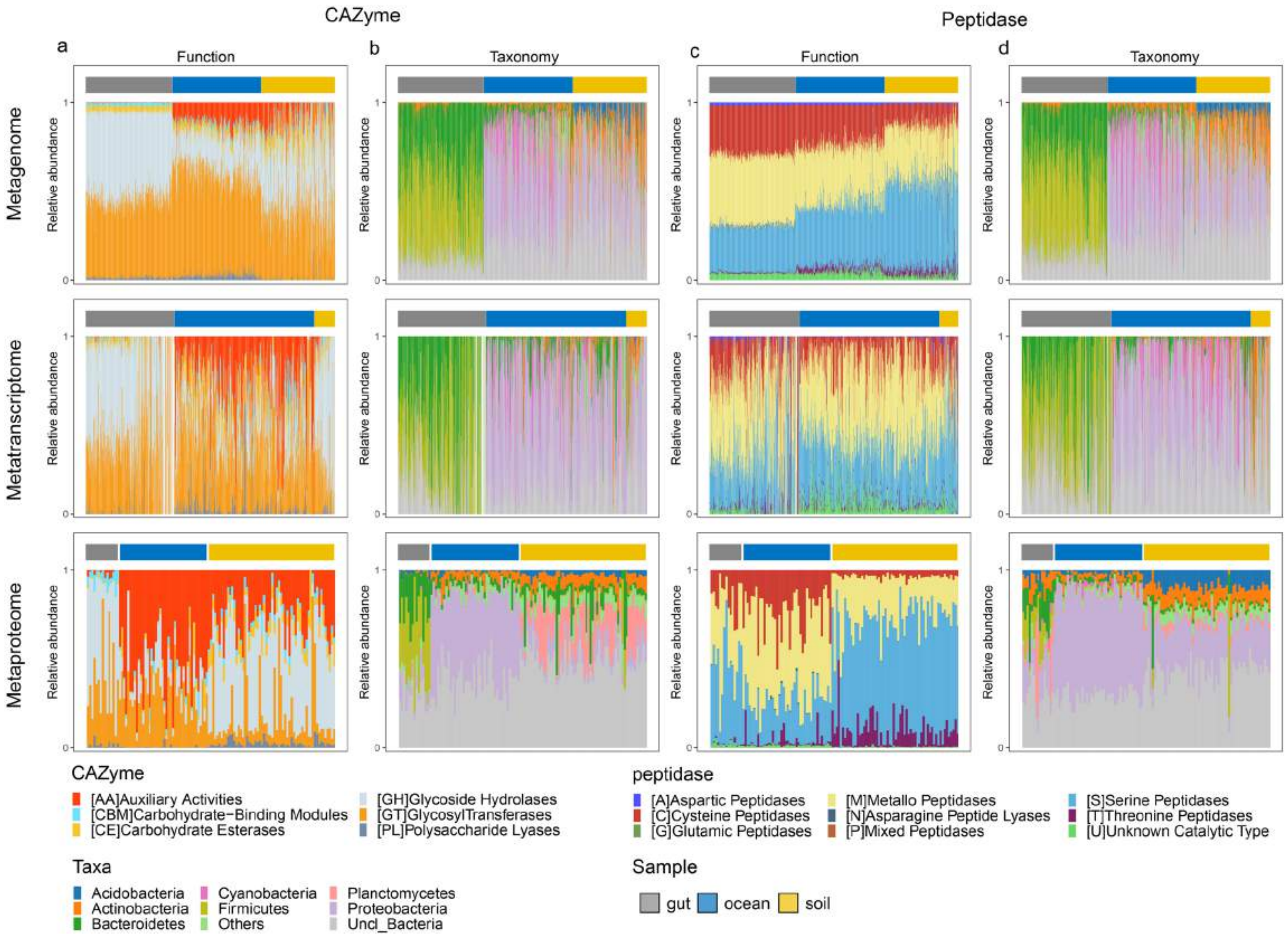

CAZyme
Peptidase
a
b
c
d
Function
Taxonomy
Function
Taxonomy
Metagenome
Metatranscriptome
Metaproteome
Relative abundance
1
0
CAZyme
[AA]Auxiliary Activities
[CBM]Carbohydrate−Binding Modules
[CE]Carbohydrate Esterases
[GH]Glycoside Hydrolases
[GT]GlycosylTransferases
[PL]Polysaccharide Lyases
peptidase
[A]Aspartic Peptidases
[C]Cysteine Peptidases
[G]Glutamic Peptidases
[M]Metallo Peptidases
[N]Asparagine Peptide Lyases
[P]Mixed Peptidases
[S]Serine Peptidases
[T]Threonine Peptidases
[U]Unknown Catalytic Type
Taxa
Acidobacteria
Actinobacteria
Bacteroidetes
Cyanobacteria
Firmicutes
Others
Planctomycetes
Proteobacteria
Uncl_Bacteria
Sample
gut
ocean
soil

Fig. S3 | Taxonomic and functional variation of cytoplasmic CAZymes and peptidases in multi-omics datasets. a, b, taxonomic and functional variation of cytoplasmic CAZymes in meta-genomic, -transcriptomic and -proteomic datasets. **c, d,** taxonomic and functional variation of cytoplasmic peptidases in meta-genomic, -transcriptomic and -proteomic datasets.

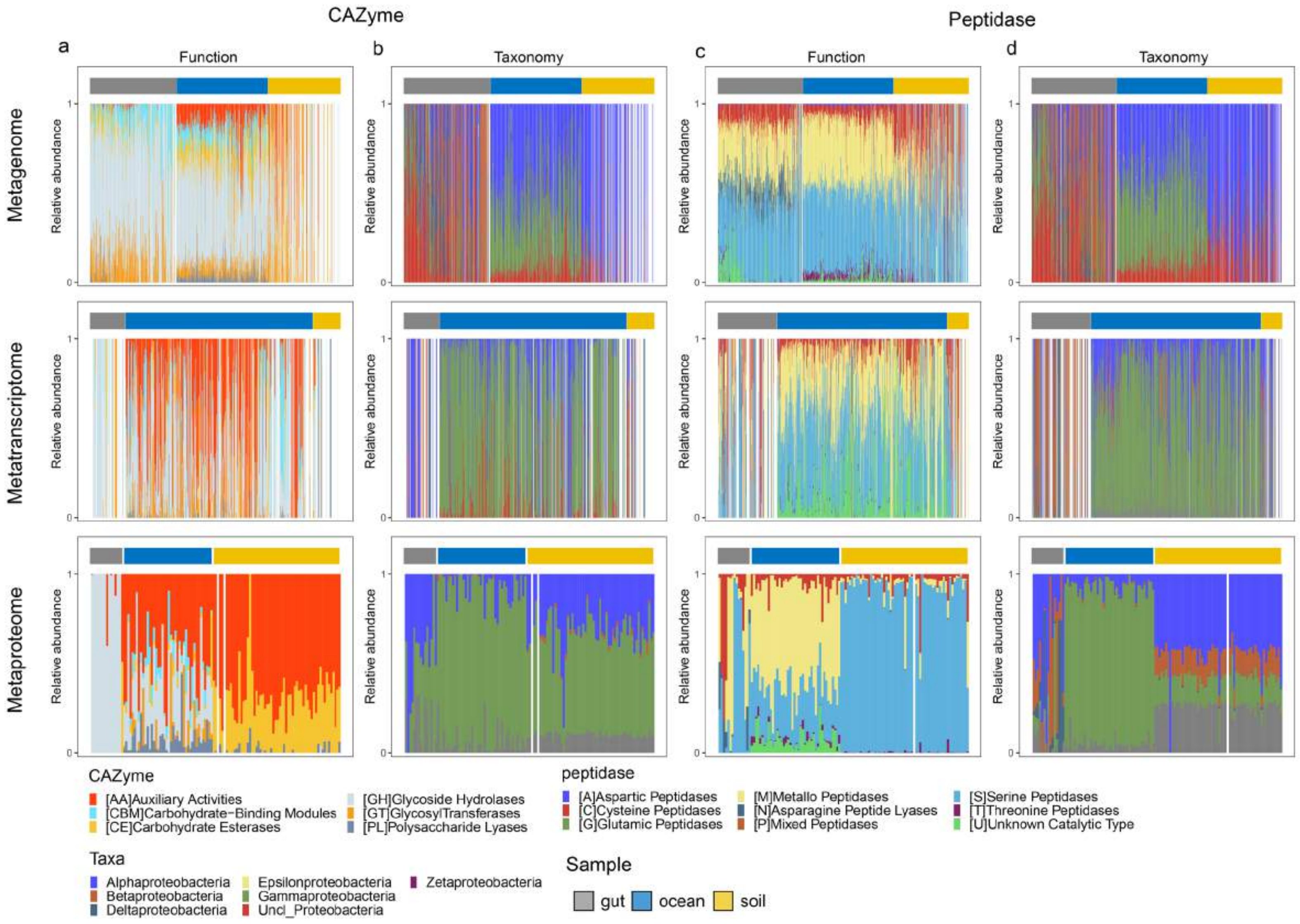
CAZyme
Peptidase
a
b
c
d
Function
Taxonomy
Function
Taxonomy
Metagenome
Metatranscriptome
Metaproteome
Relative abundance
CAZyme
[AA]Auxiliary Activities
[CBM]Carbohydrate-Binding Modules
[CE]Carbohydrate Esterases
[GH]Glycoside Hydrolases
[GT]GlycosylTransferases
[PL]Polysaccharide Lyases
peptidase
[A]Aspartic Peptidases
[C]Cysteine Peptidases
[G]Glutamic Peptidases
[M]Metallo Peptidases
[N]Asparagine Peptide Lyases
[P]Mixed Peptidases
[S]Serine Peptidases
[T]Threonine Peptidases
[U]Unknown Catalytic Type
Taxa
Alphaproteobacteria
Betaproteobacteria
Deltaproteobacteria
Epsilonproteobacteria
Gammaproteobacteria
Uncl_Proteobacteria
Zetaproteobacteria
Sample
gut
ocean
soil

Fig. S4 | Taxonomic and functional variation of Proteobacteria (at class level) affiliated secretory CAZymes and peptidases in multi-omics datasets. a, b, taxonomic and functional variation of cytoplasmic CAZymes in meta-genomic, -transcriptomic and -proteomic datasets. **c, d,** taxonomic and functional variation of cytoplasmic peptidases in meta-genomic, -transcriptomic and -proteomic datasets.

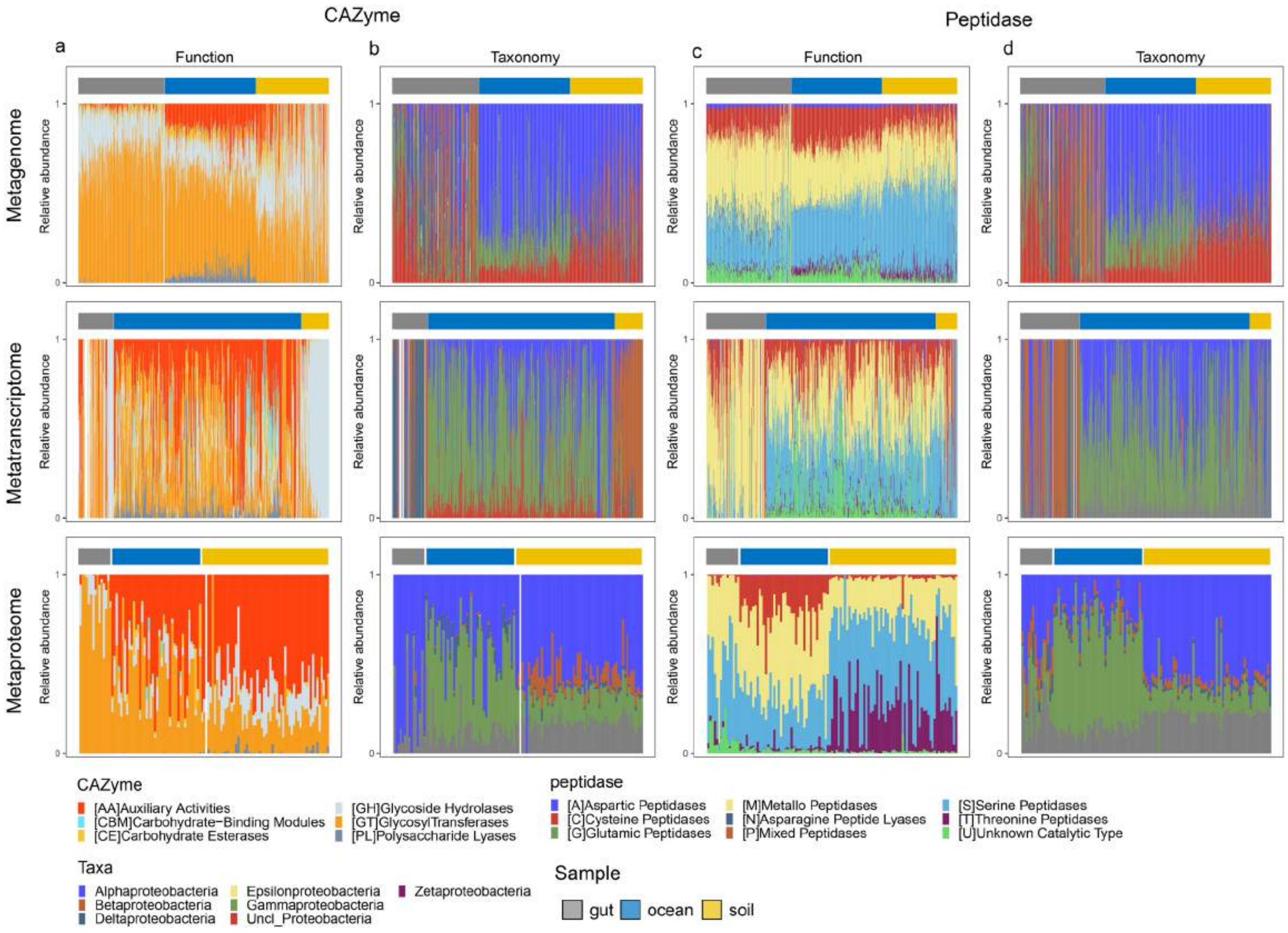
CAZyme
Peptidase
a
b
c
d
Function
Taxonomy
Function
Taxonomy
Metagenome
Metatranscriptome
Metaproteome
Relative abundance
0
1
CAZyme
[AA]Auxiliary Activities
[CBM]Carbohydrate−Binding Modules
[CE]Carbohydrate Esterases
[GH]Glycoside Hydrolases
[GT]GlycosylTransferases
[PL]Polysaccharide Lyases
peptidase
[A]Aspartic Peptidases
[C]Cysteine Peptidases
[G]Glutamic Peptidases
[M]Metallo Peptidases
[N]Asparagine Peptide Lyases
[P]Mixed Peptidases
[S]Serine Peptidases
[T]Threonine Peptidases
[U]Unknown Catalytic Type
Taxa
Alphaproteobacteria
Betaproteobacteria
Deltaproteobacteria
Epsilonproteobacteria
Gammaproteobacteria
Uncl_Proteobacteria
Zetaproteobacteria
Sample
gut
ocean
soil

Fig. S5 | Taxonomic and functional variation of Proteobacteria (at class level) affiliated cytoplasmic CAZymes and peptidases in multi-omics datasets. a, b, taxonomic and functional variation of cytoplasmic CAZymes in meta-genomic, -transcriptomic and -proteomic datasets. **c, d,** taxonomic and functional variation of cytoplasmic peptidases in meta-genomic, -transcriptomic and -proteomic datasets.

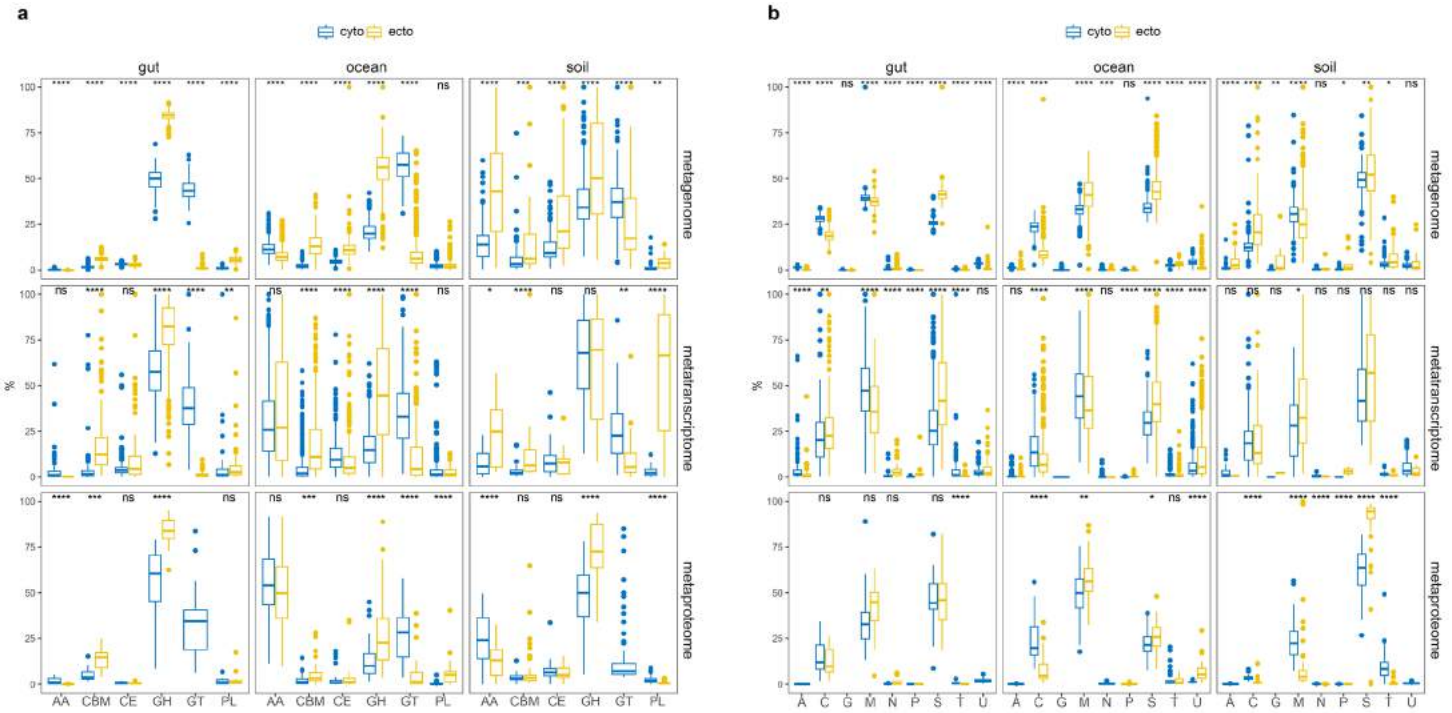
a
b
cyto
ecto
gut
ocean
soil
metagenome
metatranscriptome
metaproteome
%
AA
CBM
CE
GH
GT
PL
A
C
G
M
N
P
S
T
U

Fig. S6 | Comparison between cytoplasmic (cyto-) and secretory (ecto-) CAZymes (a) and peptidases (b) in different microbiomes.

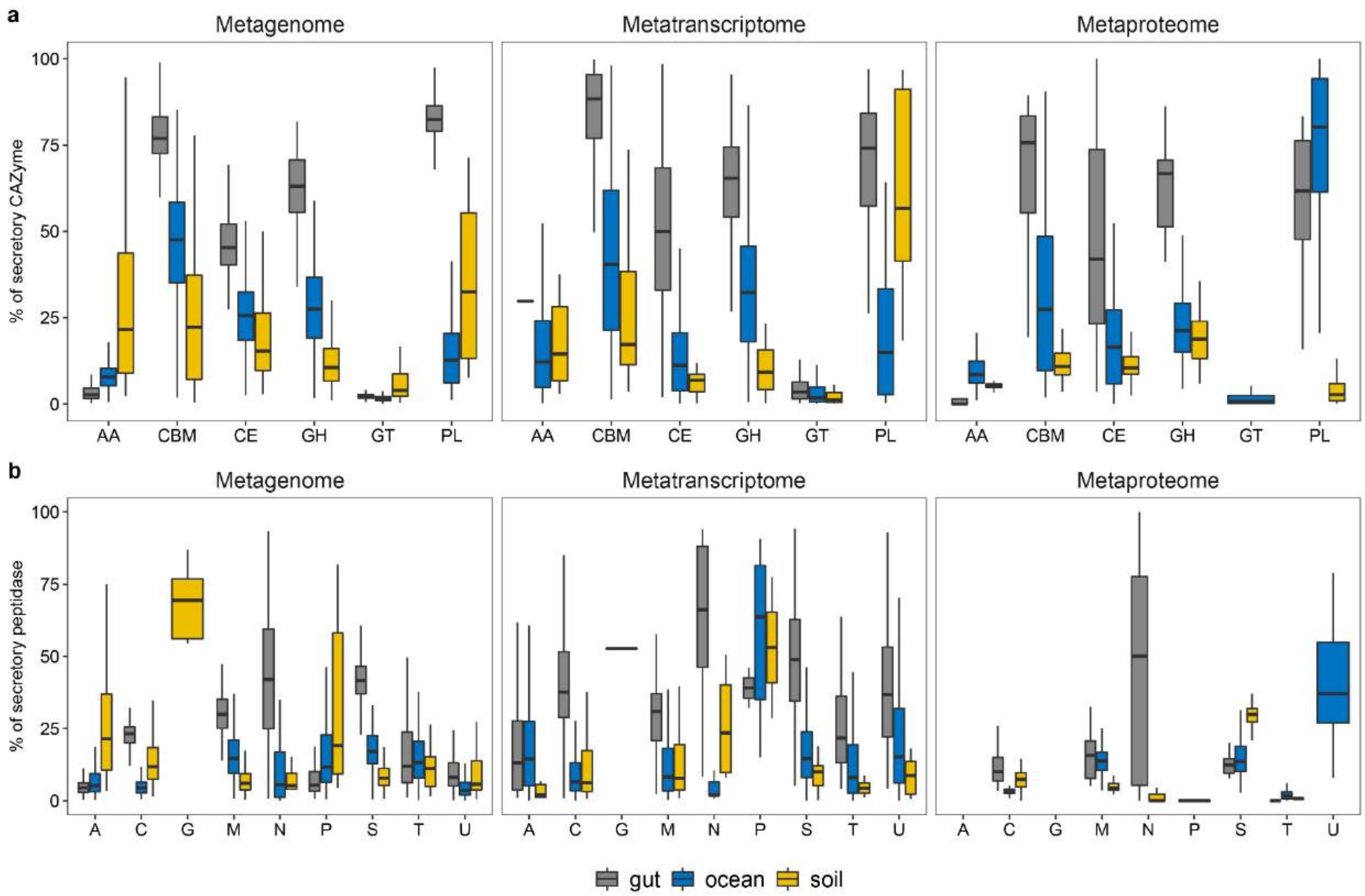
a
Metagenome
Metatranscriptome
Metaproteome
% of secretory CAZyme
100
75
50
25
0
AA
CBM
CE
GH
GT
PL
b
% of secretory peptidase
A
C
G
M
N
P
S
T
U
gut
ocean
soil

Fig. S7 | Secretory capacity (% of secretory to total) of CAZymes (a) and peptidases (b) in different microbiomes varies among functional groups and habitats.

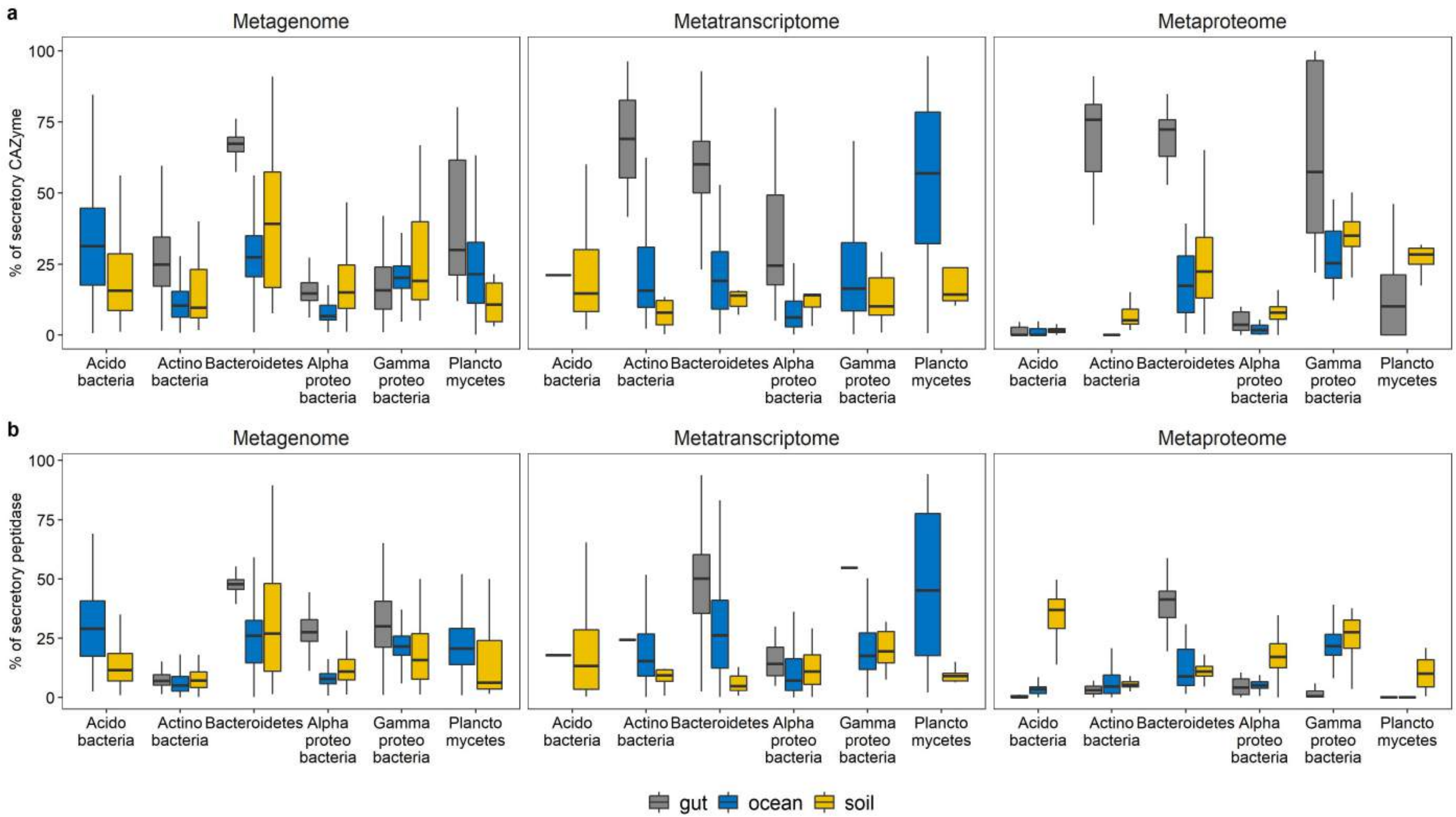
a
Metagenome
Metatranscriptome
Metaproteome
% of secretory CAZyme
100
75
50
25
0
Acido bacteria
Actino bacteria
Bacteroidetes
Alpha proteo bacteria
Gamma proteo bacteria
Plancto mycetes
b
Metagenome
Metatranscriptome
Metaproteome
% of secretory peptidase
100
75
50
25
0
Acido bacteria
Actino bacteria
Bacteroidetes
Alpha proteo bacteria
Gamma proteo bacteria
Plancto mycetes
gut
ocean
soil

Fig. S8 | Secretory capacity (% of secretory to total) of CAZymes (a) and peptidases (b) in different microbiomes varies among taxonomic groups and habitats.

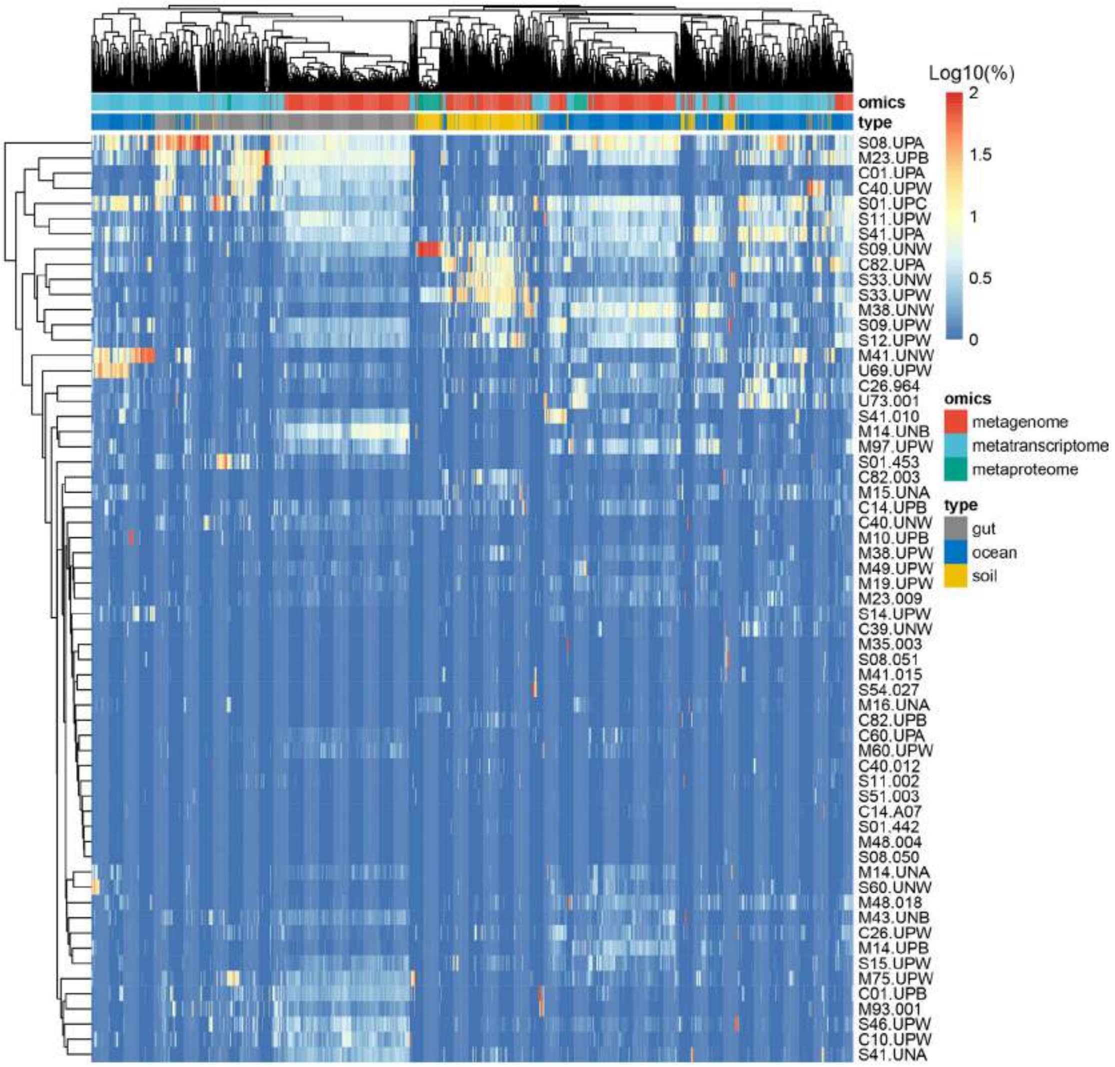

Fig. S9 | Heatmap of peptidase cleavage types in microbiomes living in the ocean, soil and human gut revealed by omics dataset.

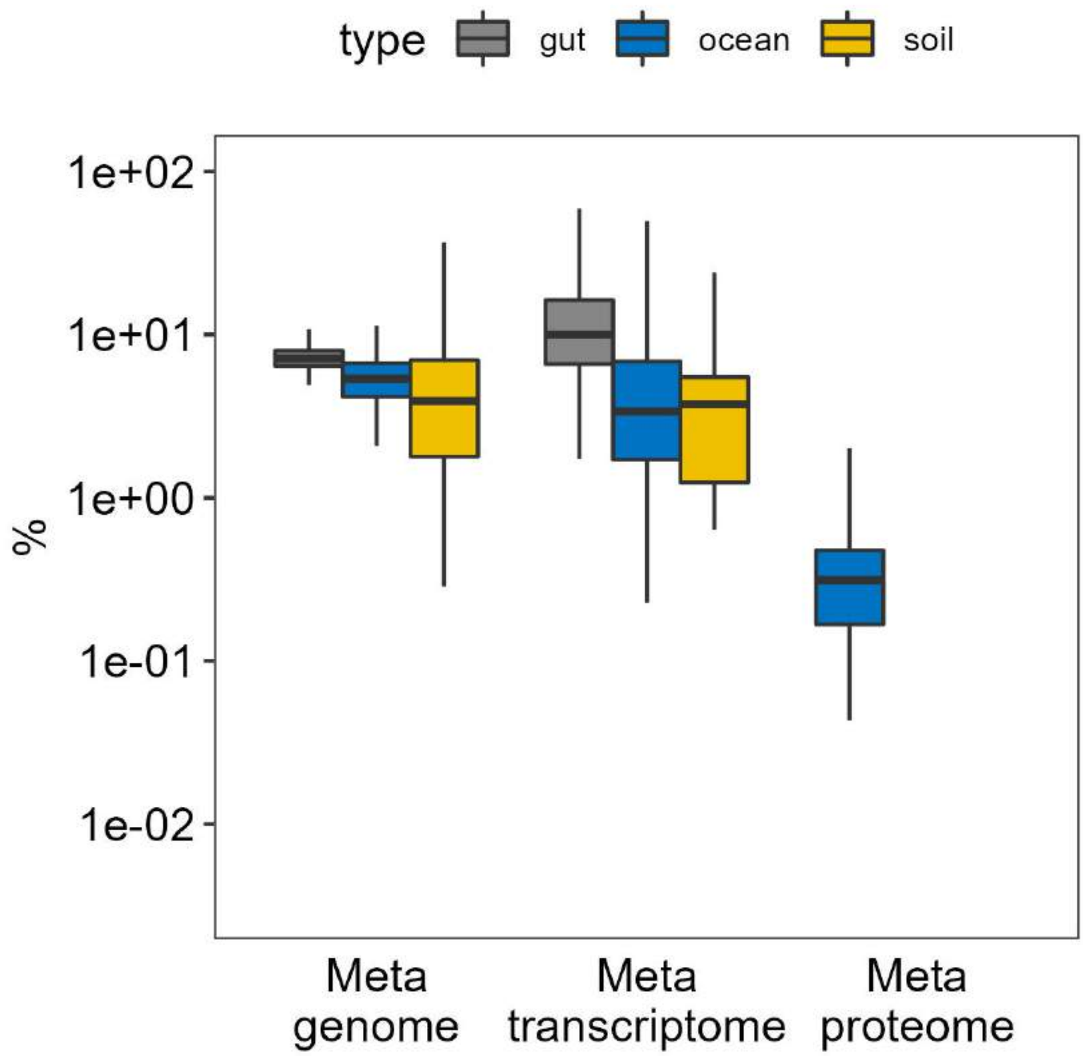

Fig. S10 | Relative abundance of M23 peptidase in multi-omics datasets.

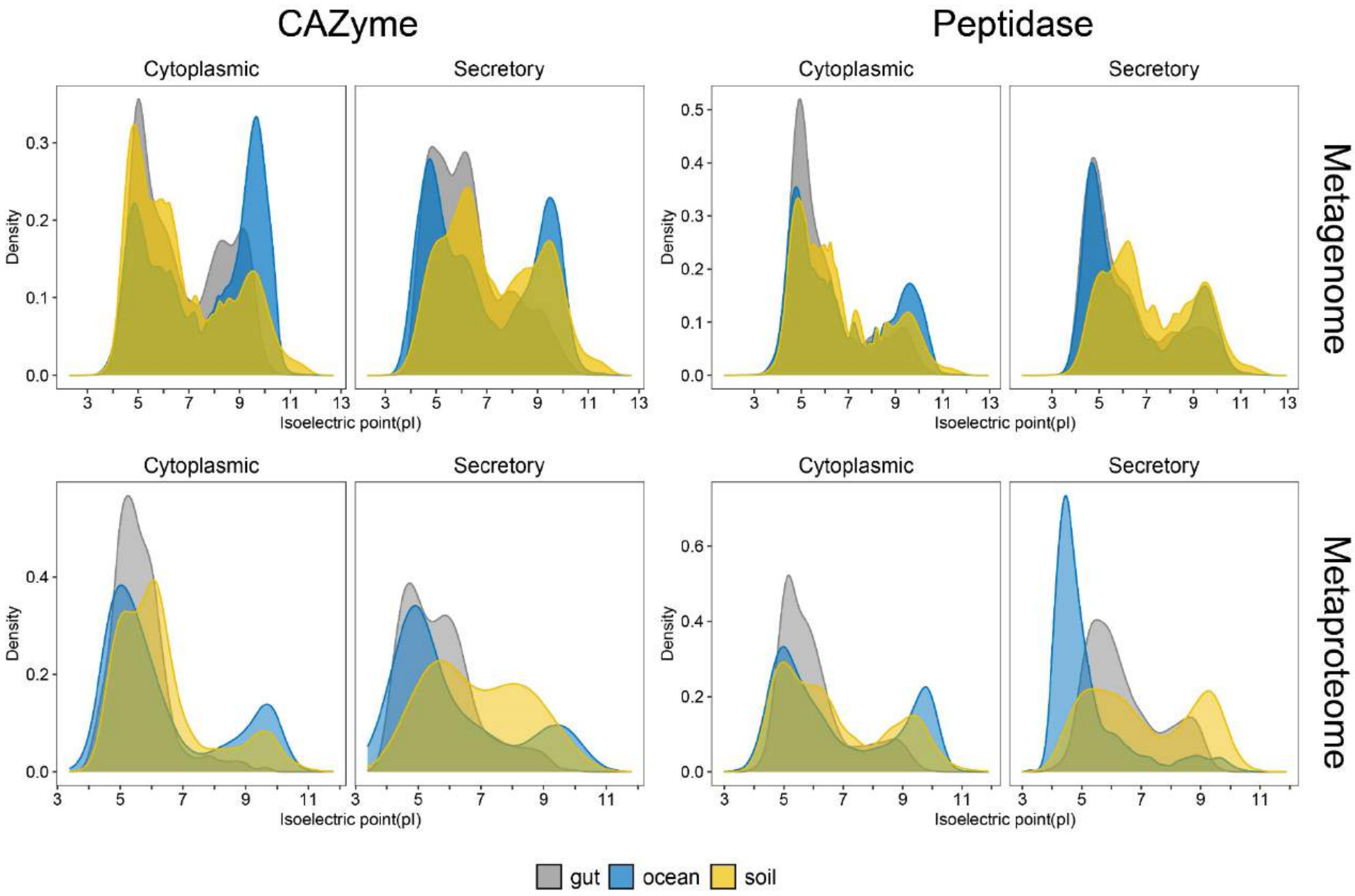
CAZyme
Peptidase
Cytoplasmic
Secretory
Cytoplasmic
Secretory
Metagenome
Metaproteome
Density
Isoelectric point(pI)
gut
ocean
soil

Fig. S11 | Isoelectric point (pI) distribution of secretory and non-secretory enzymes at the metagenome and metaproteome level.

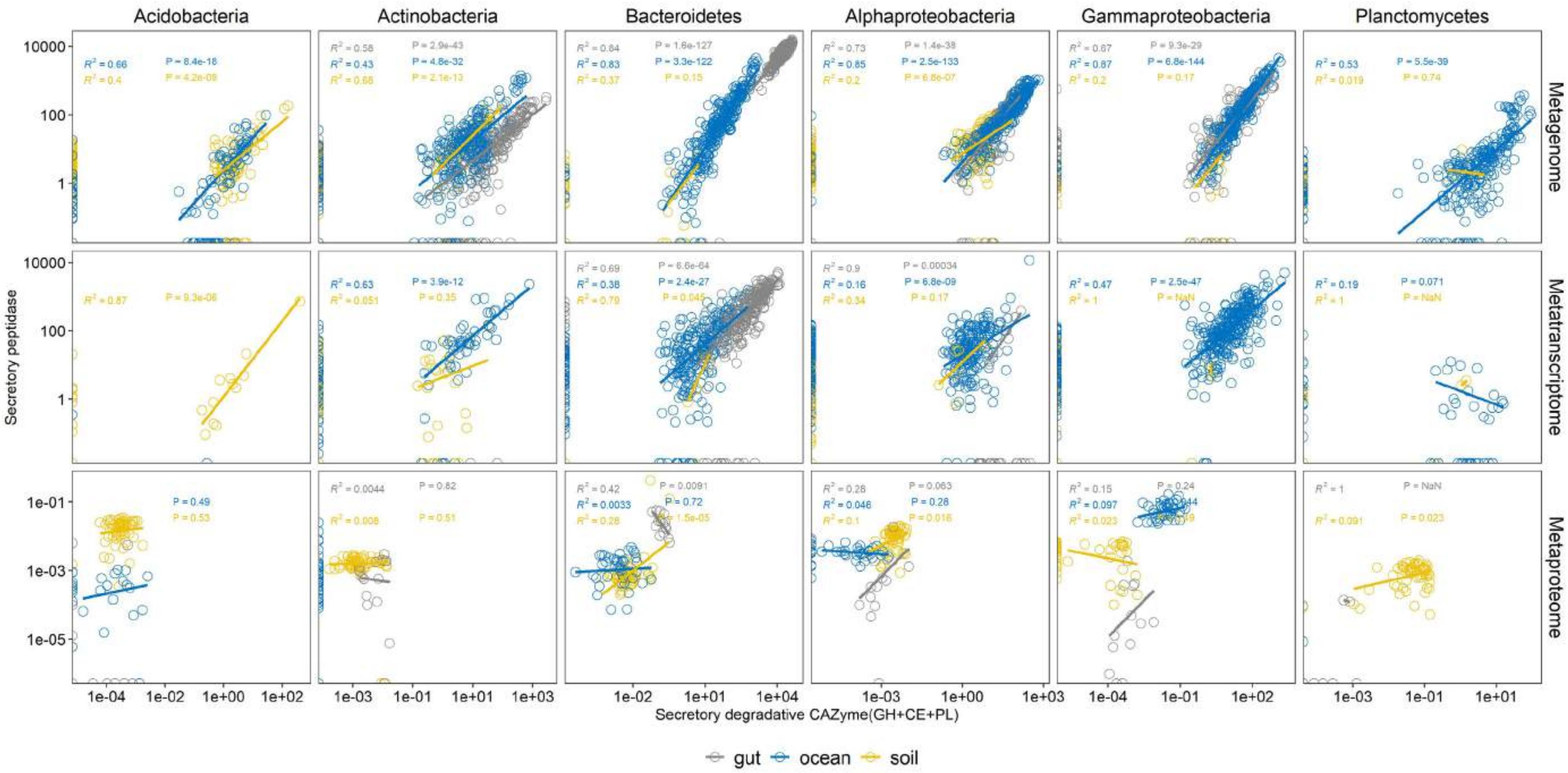
Acidobacteria
Actinobacteria
Bacteroidetes
Alphaproteobacteria
Gammaproteobacteria
Planctomycetes
Metagenome
Metatranscriptome
Metaproteome
Secretory peptidase
10000
100
1
1e-01
1e-03
1e-05
1e-04
1e-02
1e+00
1e+02
1e-03
1e-01
1e+01
1e+03
1e+04
Secretory degradative CAZyme(GH+CE+PL)
gut
ocean
soil

Fig. S12 | Relationship between the abundance of secretory degradative CAZymes and secretory peptidases in multi-omics datasets for selected taxa at the metagenome, metatranscriptome and metaproteome level.

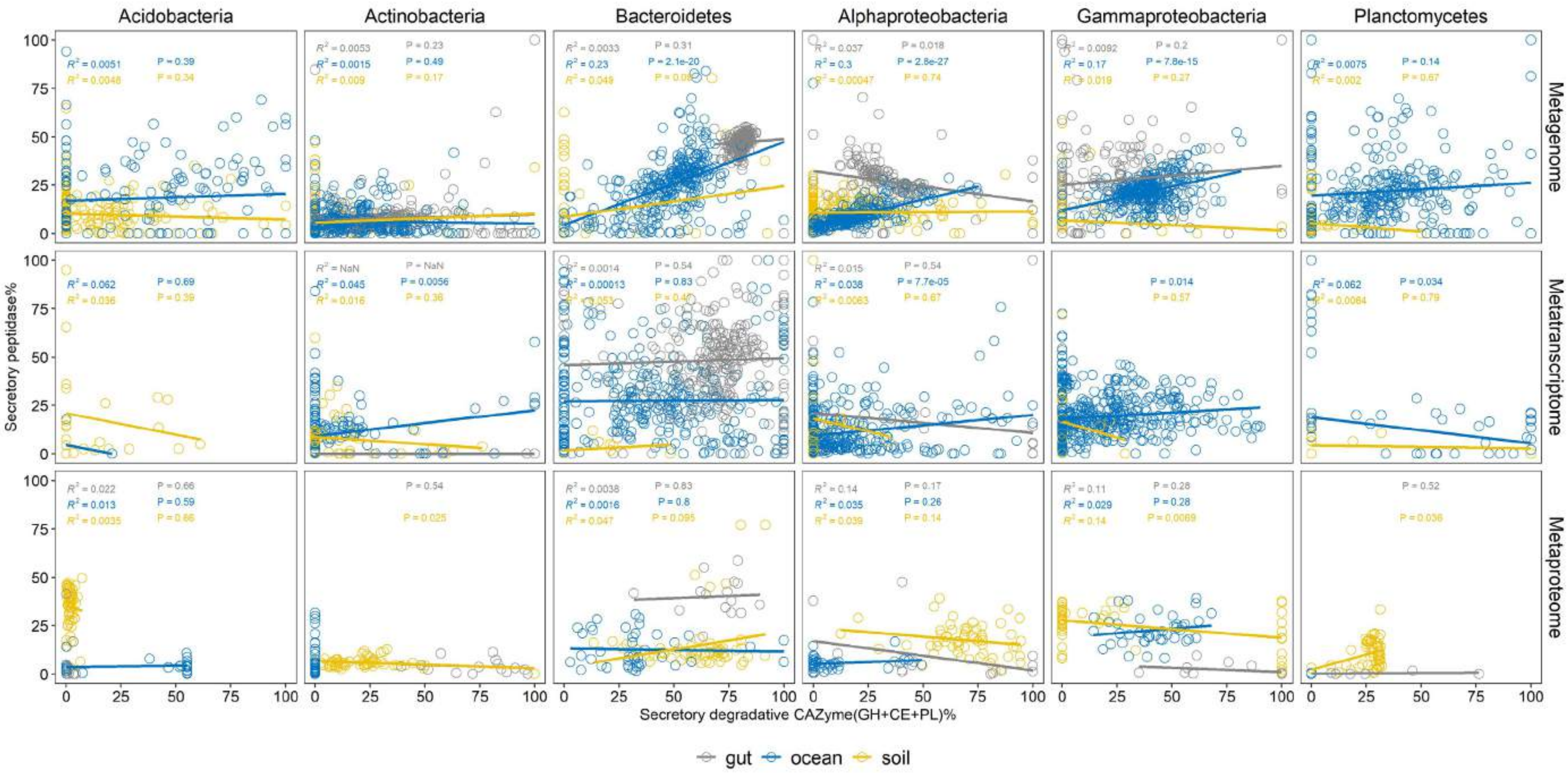
Acidobacteria
Actinobacteria
Bacteroidetes
Alphaproteobacteria
Gammaproteobacteria
Planctomycetes
Metagenome
Metatranscriptome
Metaproteome
Secretory peptidase%
Secretory degradative CAZyme(GH+CE+PL)%
gut
ocean
soil

Fig. S13 | Relationship between percentage of secretory degradative CAZymes and percentage of secretory peptidases in multi-omics datasets for selected taxa at the metagenome, metatranscriptome and metaproteome level.

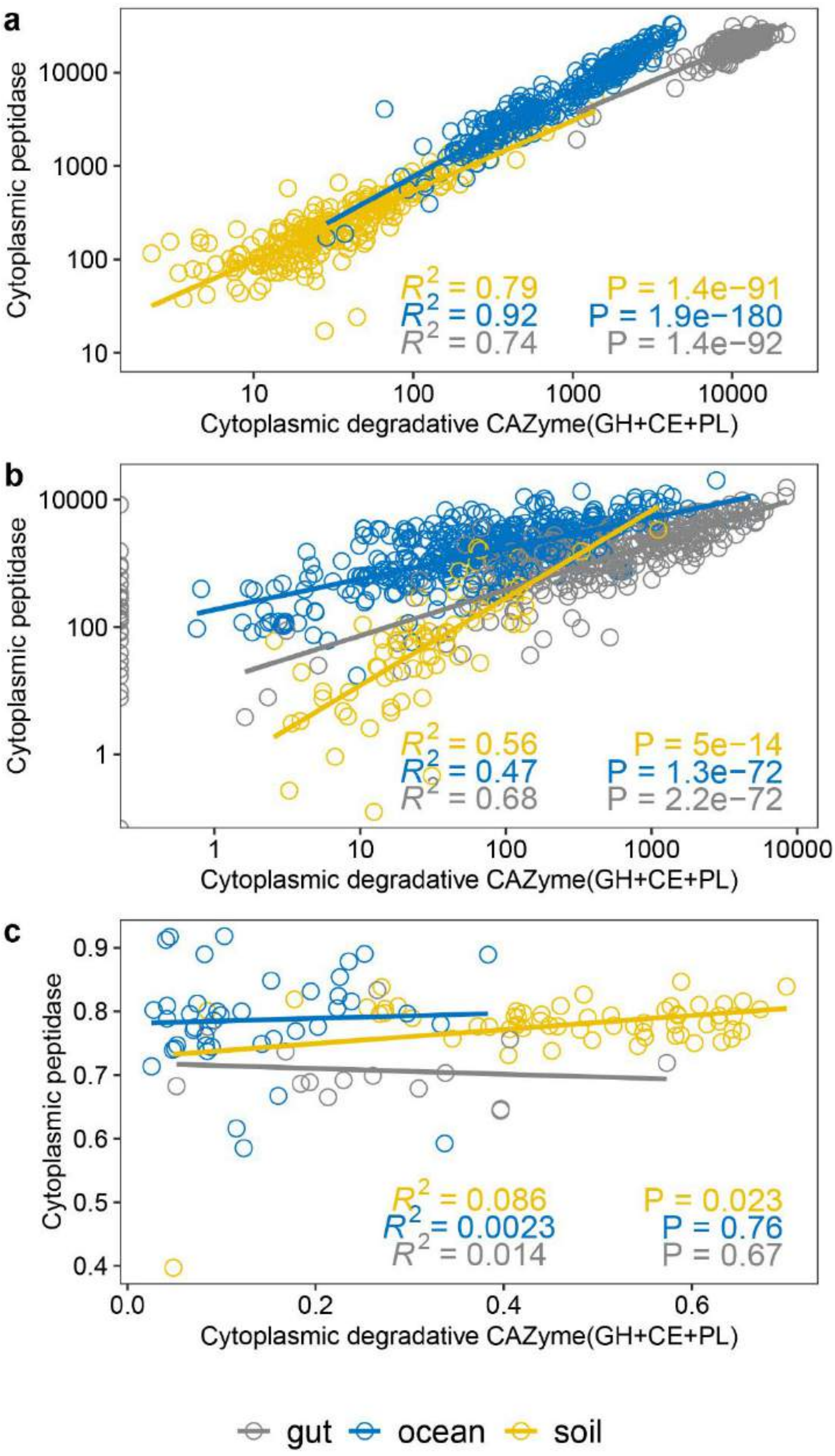
a
Cytoplasmic peptidase
10000
1000
100
10
10
100
1000
10000
Cytoplasmic degradative CAZyme(GH+CE+PL)
R2 = 0.79
R2 = 0.92
R2 = 0.74
P = 1.4e−91
P = 1.9e−180
P = 1.4e−92
b
Cytoplasmic peptidase
10000
100
1
1
10
100
1000
10000
Cytoplasmic degradative CAZyme(GH+CE+PL)
R2 = 0.56
R2 = 0.47
R2 = 0.68
P = 5e−14
P = 1.3e−72
P = 2.2e−72
c
Cytoplasmic peptidase
0.9
0.8
0.7
0.6
0.5
0.4
0.0
0.2
0.4
0.6
Cytoplasmic degradative CAZyme(GH+CE+PL)
R2 = 0.086
R2 = 0.0023
R2 = 0.014
P = 0.023
P = 0.76
P = 0.67
gut
ocean
soil

Fig. S14 | Relationship between the abundance of cytoplasmic degradative CAZymes and cytoplasmic peptidases in multi-omics datasets. a, metagenome**. b,** metatranscriptome and **c,** metaproteome.

Table S1 Summary of genes/proteins analyzed

		Gut	Ocean	Soil
**No. of total genes**		9,878,647	487,363,790	159,657,012
**No. of CAZyme genes**	**Cytoplasmic**	170,142	3,008,787	1,169,214
	**Secretory**	49,915	314,318	103,950
**No. of peptidase genes**	**Cytoplasmic**	183,555	8,806,760	3,192,236
	**Secretory**	38,329	896,494	291,929
**No. of CAZymes**	**Cytoplasmic**	7,031	29,341	64,706
	**Secretory**	5,904	4,557	13,175
**No. of peptidases**	**Cytoplasmic**	13,488	241,252	138,979
	**Secretory**	2,914	32,156	24,001

Table S2 Summary of omics datasets used

	Gut	Ocean	Soil
**No. of metagenomes**	316	326	278
**No. of metatranscriptomes**	336	519	72
**No. of metaproteomes**	8	49	60
**Description**	The stool samples were collected from citizens living in Denmark, Spain and USA	Water samples were collected from major ocean basins covering epi- (<200m), meso- (200-1000m) and bathy-pelagic (>1000m) waters	Soil samples were collected from surface soils (<5cm) covering a variety of types ranging from Arctic tundra to moist tropical forests

Table S3 CAZymes targeting different substrates

Substrate	CAZyme
Plant Carbohydrates	GH1;GH2;GH3;GH4;GH5;GH8;GH9;GH11;GH12;GH15;GH16;GH17;GH26;GH27;GH28;GH29;GH36;GH39;GH43;GH44;GH48;GH51;GH53;GH55;GH67;GH74;GH78;GH93;GH94;GH95;GH115;GH117;GH121;PL1;PL2;PL6;PL7;PL9;PL11;PL15;PL22
Animal Carbohydrates	GH1;GH2;GH3;GH4;GH18;GH19;GH20;GH29;GH33;GH38;GH58;GH79;GH84;GH85;GH88;GH89;GH92;GH95;GH98;GH99;GH101;GH105;GH109;GH110;GH113;PL6;PL8;PL12;PL13;PL21
Peptidoglycan	GH23;GH24;GH25;GH73;GH102;GH103;GH104;GH108
Algae Carbohydrates	GH16; GH50; GH55;GH86;GH118; GH64;GH158;GH16;PL7;PL14;PL15;GH82;PL24;PL25;PL28;PL40
